# Functional Imaging of the Foot with Perfusion Angiography in Critical Limb Ischemia

**DOI:** 10.1007/s00270-015-1253-6

**Published:** 2015-12-01

**Authors:** Jim A. Reekers, Mark J. W. Koelemay, Henk A. Marquering, Ed T. van Bavel

**Affiliations:** Dept of Radiology, AMC, Meibergdreef 9, 1105 AZ Amsterdam, The Netherlands; Dept of Vascular Surgery, AMC, Meibergdreef 9, 1105 AZ Amsterdam, The Netherlands; Dept of Biomedical Engineering and Physics, AMC, Meibergdreef 9, 1105 AZ Amsterdam, The Netherlands; Department of Radiology G1.206, Academic Medical Center, University of Amsterdam, Meibergdreef 9, PO box 22660, 1100 DD Amsterdam, The Netherlands

**Keywords:** Experimental IR, Imaging, Clinical practice, Arterial intervention, Diagnostic, Arteriosclerosis, Diabetes, Ischemia

## Abstract

**Purpose:**

To report on the first clinical experience with perfusion angiography (PA) of the foot in patients with chronic critical limb ischemia.

**Materials and Methods:**

PA is a post-processing software algorithm and no extra digital subtraction angiography (DSA) has to be performed for this analysis. The data used to test the feasibility of PA were obtained from a consecutive group of 89 patients with CLI who were treated with standard below the knee angioplasty and 12 separate patients who were not suitable for endovascular revascularization.

**Results:**

Motion artifacts in the dataset of the DSA made post-procedural analysis impossible in 10 % intervention. In the majority of patients (59/68) PA showed an increase in volume flow in the foot after successful angioplasty of the crural vessels. However, in 9/68 patients no increase was seen after successful angioplasty. With the use of a local administered competitive α-adrenergic receptor antagonist, it is also possible to test and quantify the *capillary resistance index* which is a parameter for the remaining functionality of the microcirculation in CLI patients.

**Conclusion:**

PA might be used as a new endpoint for lower limb revascularization and can also be used to test the functionality the microcirculation to identify sub-types of patients with CLI. Clinical evaluation and standardization of PA is mandatory before introduction in daily practice.

## Introduction

Local perfusion problems in the foot, like a non-healing ulcer, are often caused by macrovascular obstructions, sometimes in combination with disease at the level of the microcirculation of the foot. The latter is especially true for arterial diabetic foot disease. Endovascular revascularization of the macrovessels is the first-line treatment; however, there are no validated tests to predict the clinical outcome after successful revascularization and often only time will tell. Also a technically successful revascularization on digital subtraction angiography (DSA) is not always also a predictor for good outcome and vice versa. Any increase in volume flow to the foot will only be clinically beneficial when this also translates into a sufficient perfusion of the capillaries and subsequent tissue oxygenation of the foot. Newly emerging techniques, especially those based on magnetic resonance imaging, allow mapping of areas of poor tissue oxygenation and perfusion of the whole foot, beyond the skin [1]. These techniques are still under development but moreover they are not instantly available during a revascularization procedure for decision making.

Perfusion angiography (PA), which is a representation of the time-density curve of contrast volume flow in the foot, is a new imaging technique, using data from plain old digital subtraction angiography, for assessment of foot perfusion [2]. Studies to show the clinical validity of PA are underway but will still take 2 years to be finished.

Because the PA software (Philips medical, Eindhoven, The Netherlands) application is already commercially available, we think it is important for the understanding and standardization of PA to report our initial feasibility data.

## Materials and Methods

### Patients

The data used to test the feasibility of PA were obtained from a consecutive group of 89 patients, all with CLI according to the European consensus document, who were treated with standard below the knee angioplasty at our institution during the last 18 months. These patients represent the normal CLI population with a broad scale of underlying pathology like diabetes, atherosclerosis, M Burger, and peripheral embolization. A separate group of 12 patient had, based on the extension of the disease and the anatomy during DSA, no option for endovascular treatment, and therefore the procedure was terminated after diagnostic angiography.

*Design* Prospective observational data to test the feasibility of PA.

All procedures performed in studies involving human participants were in accordance with the ethical standards of the institutional and/or national research committee and with the 1964 Helsinki declaration and its later amendments or comparable ethical standards.

Because no extra imaging was needed and the data were not used in a clinical setting and no patients related demographics were included in this first feasibility study, informed consent was waved by our local medical ethical committee.

### Technique

The software for analyzing the perfusion is developed by Philips medical (Best, The Netherlands) and is based on the calculation of the change in density per pixel over time. For post-procedural quantification of the perfusion with PA the original DSA datasets obtained during angioplasty are used. PA is a post-processing software algorithm and no extra DSA has to be performed for this analysis. The calculation was performed only after the procedure was finished and the PA data were not used for any decision making.

In this group in which angioplasty was not performed, vasodilation with tolazoline (Novartis, Basel Suisse) was used as part of the diagnostic work-up procedure.

**Fig. 1:**
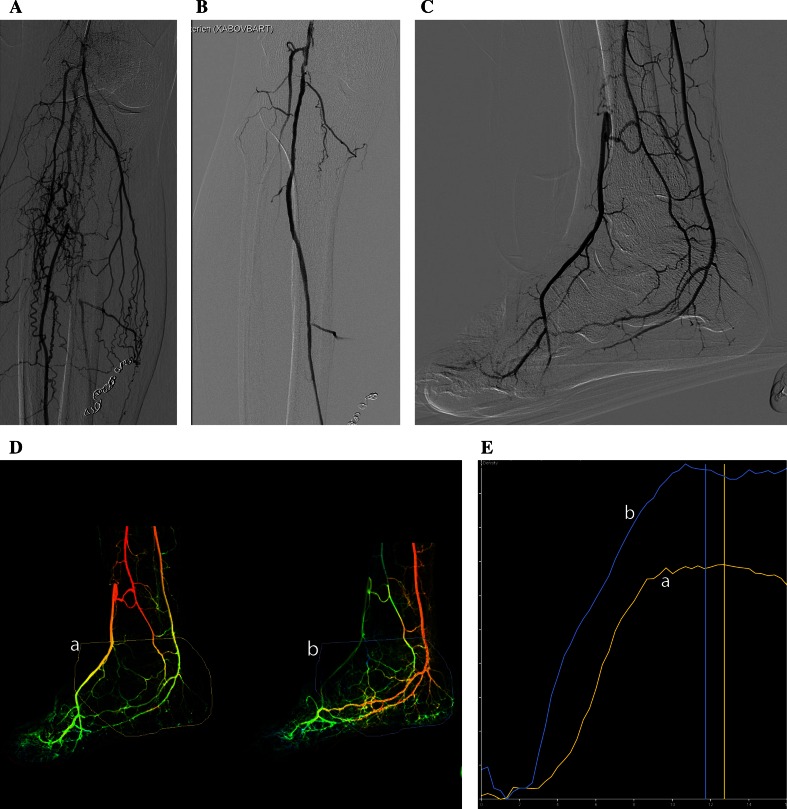
*CLI* no diabetes and non-healing ulcer on the foot. **A** Occlusion of the trifurcation. **B** Revascularization of the posterior tibial artery, the only artery with direct outflow to the foot. **C** Outflow in the foot. **D** Perfusion angiography pre- intervention (*A*) and post–intervention (*B*) with ROI. **e** Strong improvement of flow after revascularization. The area under the curve increased with 48 % and the maximal density peak with 40 %

A standardized DSA protocol including lateral foot projection was used in all patients pre- and post-crural angioplasty. After selective ipsilateral femoral catheterization and, if necessary, endovascular optimization of the flow in the above the trifurcation arteries, but before crural angioplasty, a 5 Fr end-hole catheter is positioned at the level of the mid-popliteal artery. Nine ml of Visipaque 320 mg I/ml (Iodixanol, GE healthcare, Cork Ireland) is injected at a speed of 3 ml/s with 900 psi. The DSA is made with the foot in lateral projection [2]. Because with the current software any motion artifacts in the dataset of the DSA will make post-procedural analysis impossible, the foot is fixed in a dedicated footrest. DSA images are obtained at a frame rate of 3/s. During 15–20 s. Change in volume flow was measured as change in area under the curve and change maximal peak density. To obtain a flow-density curve on the workstation, a box was manually placed over the region of the foot, not lower than the middle cuneiform bone, where the arterial foot arch is situated (Fig. 1A). With the available software, the starting point of the time-density curves, pre- and post-revascularization images, can be matched. The difference in pre- and post-intervention perfusion is expressed in change in area under the curve and change in maximal peak density. The area under the curve is measured from the starting point of the curve until the maximal peak density in one of the two curves has been reached. The change in perfusion is calculated in percentage in change in the area under the curve (Fig. 1). The change in maximal peak density is calculated in percentage increase or decrease. (Fig. 1).

## Results

10 % (9/89) of the DSA datasets, pre- or post-angioplasty, were not useful for PA analysis because of motion artifacts. In 12 patients, crural angioplasty was not performed due to the local anatomy or already major angiographic flow improvement after an above-the-knee intervention. 68 patients were analyzed pre- and post-crural intervention. In the majority of patients (59/68), PA showed an increase in volume flow, both increase in area under the curve and maximal peak density, in the foot after successful angioplasty of the crural vessels. This improvement ranged from <10 to 40 % for maximal peak density and from <10 to >100 % for area under the curve. The average increase after successful pta was 21 % for maximal peak density and 48 % for area under the curve. In 9/68 patients, no increase was seen after an angiographical successful angioplasty. After revascularization, the increase in volume flow was different for each individual patient and did not show any direct relation to the original DSA appearance pre- and post-intervention. Due to technical observational design of this study, without patient informed consent, we were not allowed to link PA changes to the clinical outcome.

## Pharmacological Stimulation

Vasodilation was used in the 12 patients without options for a revascularization procedure below the knee. Tolazoline is a non-selective competitive α-adrenergic receptor antagonist that gives a twofold to fivefold increase in A-V shunting at capillaries level. Tolazoline, (4 mg) was diluted in 10 ml saline and was injected through the catheter with the tip mid-popliteal. The delay after tolazoline to perform a new DSA was 60 s. The response to tolazoline is an instant first pass response after injection, which can maintain up to 40 min. The response to tolazoline can be quantified with the available software and was calculated as the index, post divided by pre, of change in maximal peak density. An index of 1 is equal to no change.

## Results

**Fig. 2:**
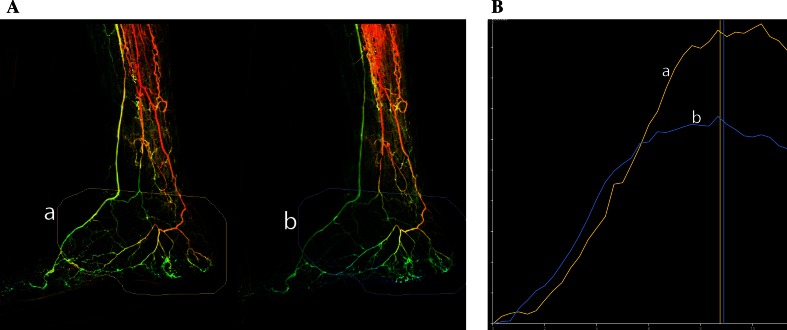
Diabetes and CLI and non-healing ulcer on the foot. Posterior tibial artery occluded, stenosis (50 %) in anterior tibial artery. **A** Initial angiography (ROI A) and angiography after tolazoline (ROI B). **B** Faster flow with early maximal density peak after tolazoline. The calculated capillary resistance index is 0.6. Follow-up: conservative treatment with good healing of the ulcer no amputation

**Fig. 3:**
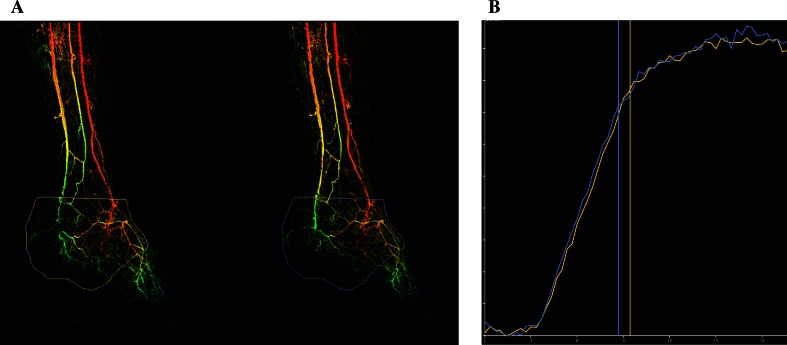
Diabetes and CLI and non-healing ulcer on the foot. Three tibial arteries are open with collateral outflow in the foot. **A** Initial angiography (*left*) and angiography after tolazoline (*right*). **B** There is no change in flow through the capillaries, both curves pre- and post-tolazoline overlap. Calculated capillary resistance index is 1. Conservative treatment. Follow-up: fast decline of the ulcer and early amputation

PA after tolazoline showed a faster passage of contrast in 7/12 patient which resulted in an earlier and lower maximal density peak (Fig.  2). The average index was 0.7. Five 5 patients however did not show any response to tolazoline (Fig. 3). The average index was 1 (±0.1). In these patients, the perfusion curves pre- and post-tolazoline overlapped. All 5/12 patients who showed no response had diabetic foot disease, while from the seven responders, 4/7 had atherosclerosis and 3/7 diabetic foot disease. There is a 100 % correlation between non-responders and diabetic foot disease.

## Discussion

The microcirculation of the foot includes the terminal arterioles and capillaries beyond the arteries that are involved in transportation of the oxygen and blood nutrients to the tissue.

Transport of oxygen to the tissue of the foot requires blood flow through the capillaries and subsequent diffusion of oxygen to the tissue. The capillary volume flow is pressure dependent but capillary pressure cannot be measured directly and local arterial pressure in the pre-capillary vessels (e.g., toe pressure) is not always proper proxy for arterial insufficiency in the foot [3]. Subsequent transport of oxygen from the capillaries to the tissue is by diffusion and is not pressure dependent. Decrease in microcirculation perfusion is therefore equal to decrease in available oxygen for tissue oxygenation. Many patients with CLI, even without calcified vessels, have a toe pressure above the consensus document threshold for critical limb ischemia [4–6]. Toe pressure is however a parameter of the macrocirculation. These are patients where macrovascular blood volume flow to the foot is sufficient, but the flow through the microcirculation (perfusion in the foot)—and thus oxygen transport—is insufficient. As there is a direct relation between flow through the capillaries and the oxygenation of the tissue, a direct measurement of flow through the capillaries, pre-and post-intervention, with PA might be a good parameter for improved foot perfusion and tissue oxygenation.

Until now all focus in CLI has been on the ulcer and the ulcer healing. We think that the ulcer, and moreover the non-healing ulcer, is a symptom of a general diseased microcirculation of the foot. The first paradigm shift is that not only the area of the ulcer is diseased but the whole foot and that the general underlying microvascular pathology will finally determine clinical outcome and not merely the local perfusion improvement in the ulcer. Therefore, PA looks at the overall perfusion of the foot and not merely at ulcer region perfusion, because a non-healing ulcer is often only a symptom of general microcirculation pathology. The relation between inflow (macrocirculation) and capillary flow, which is directly related to oxygenation of the tissue of the foot, is very important for ulcer healing. An optimal inflow with a poor capillary flow will lead to low tissue oxygen and consequently poor ulcer healing. Visa versa, a low inflow volume with a healthy microcirculation will clinically often show no CLI but only intermittent claudication. This is also an explanation for the often contradictory finding that in the first scenario vascularization on angiography can sometimes be surprisingly good, while in the other scenario the angiography can show poor vascularization. This often poor relation between angiographic appearance and clinical presentation is well known in daily practice. Both the PA (capillary flow) respond to crural revascularization as the PA respond to specific stimulation of the microcirculation with tolazoline contains important functional information about the microvascular functionality of the foot. This functionality of the microcirculation could probably be a better predictor for outcome than, e.g., a technical successful revascularization.

The foot consists of >90 % microcirculation and <10 % macrovessels. Therefore, the role of the macrocirculation in the PA calculations is, although not zero, small. The flow calculations are therefore only minimally influenced by the macrovessels. However, in patients without intervention or calculations pre-intervention this plays no role. This minimal relation between macro- and microvascularisation is also reflected in the clinical experience where it is known that improving the inflow to the foot is not always a guarantee for a good clinical outcome [7–11]. The outcome of CLI is very unpredictable, as is shown in the paper by Lepantalo et al. [10]. This retrospective study evaluated 105 consecutive patients with 136 critically ischemic legs as defined by the European Consensus Document on Chronic Critical Leg Ischemia, who had no options for revascularization. Fifty-four percent of these patients did not have an amputation at the 12-month follow-up, and this result was the same for diabetic and non-diabetic patients. On the other hand, amputation despite successful revascularization is also known. Even with a patent infrainguinal lower extremity bypass for CLI, about 10 % of patients do not achieve clinical improvement at 1-year follow-up [8–11].

Based on our initial experience with PA, we think that the area under the curve and the maximal peak density are both parameters of the total blood volume passing through the foot (micro- and macrocirculation) over time. The length of the curve (=time) is of less importance and the plateau phase is probably more important. We hypothesize that in CLI a significant increase in volume flow on PA, after a revascularization procedure, might be a predictor for good outcome, while minimal or no increase might be herald a bad outcome. This creates the opportunity, as this is a instantly available tool during endovascular treatment, for opening more vessels or outflow before ending a procedure to obtain optimal inflow. The percentage in volume flow difference between pre- and post-revascularization can therefore be used as a new endpoint for (crural) revascularization in CLI. However, the true cut-off point for a successful revascularization, based on PA, still needs to be determined in a prospective clinical study, which is currently underway.

Tolazoline is a non-selective competitive α-adrenergic receptor antagonist that has the same effect as lumbal sympathectomy, only short term. Like with sympathectomy it widens the capillary resistance vessels and local administration of tolazoline in the foot gives a twofold to fivefold increase in A-V shunting at capillaries level, but does not fully override or blocks the natural vasoconstrictive activity of the capillary sphincter [12, 13]. Tolazoline has less effect on the afferent arterioles [12]. Tolazoline works different from nitroglycerin or any other vasodilating drug, which mainly induces dilatation of the macrocirculation and collaterals and can therefore not be used for functional imaging. Pharmaco-stimulation of the microcirculation is a test to measure the remaining functionality. When there is no response to tolazoline, there is probably also little chance that any extra inflow of blood after revascularization will be contributing to the tissue oxygenation. Because this is a functionality test, we presume that the quantification of response to pharmacological stimulation should better be calculated as an index; the *capillary resistance index* (CRI). The equation is maximal peak density after stimulation divided by the maximal peak density pre. This index is automatically calculated by the software on the workstation after manual placement of the vertical lines (cursors) on the calculated perfusion curves (Fig. 2). If there is a normal response with increase in capillary AV-shunting after tolazoline, this results in a faster passage time of the standardized contrast bolus and subsequently a lower peak density The calculated CRI will subsequently be <1 (Fig. 2). However, if the AV-shunting does not increase, which is equal to absence of a good functional response of the microcirculation, the pre- and post-perfusion will be the same, and the CRI will be around 1. This was seen in five diabetes patients in our feasibility study (Fig. 3). The CRI might therefore be a new parameter to test the degree of microcirculation disease and to differentiate between diseases with or without microcirculation involvement, like in diabetic and atherosclerotic foot disease. Unlike with measuring volume flow after revascularization the change in volume flow during functional imaging with pharmaco-stimulation is not equal to absolute perfusion flow but only a parameter for the functionality of the microcirculation.

CRI might also be a test to select patients who will have a good outcome after revascularization and those who will not. The latter also has huge implication regarding costs of treatment.

Although PA looks very promising for functional imaging of the foot in CLI, it will only be clinically valuable with meticulous standardization and after proper clinical evaluation. The standardization of the technique and the reporting of outcome as discussed in this paper is therefore absolutely mandatory for the success of PA.

In conclusion, our first experience to test the feasibility of PA shows that the increase in volume flow in the foot after revascularization can be instantly measured and quantified with PA, and that this might be an important new endpoint for any endovascular revascularization procedure. This endpoint could also hold important predictive information about outcome, but this has to be investigated in a prospective observational clinical trial with clinical endpoints. Using a non-selective competitive α-adrenergic receptor antagonist, it is possible to quantify the functionality of the microcirculation in patients with CLI. The CRI of the foot might allow for identification of different sub-types of clinical and angiographic identical CLI patients. PA might give important information about the functionality of the microcirculation in CLI patients.
